# Supplementary material for: Antibiotic and Nematocidal Metabolites from Two Lichen Species Collected on the Island of Lampedusa (Sicily)
Source: Int J Mol Sci. 2022 Jul 30;23(15):8471. doi: 10.3390/ijms23158471 (PMC9368951; doi:10.3390/ijms23158471)
Supplement: Supplementary file 1 [file ijms-23-08471-s001.zip › ijms-1830208-supplementary.pdf]

International Journal of *Molecular Sciences*

MDPI

Antibiotic and nematocidal metabolites from two lichen species collected on the island of Lampedusa (Sicily)

Jesús García Zorrilla[1,2],* Trifone D'Addabbo[3], Emanuela Roscetto[4], Chiara Varriale[4], Maria Rosaria Catania[4], Maria Chiara Zonno[5], Claudio Altomare[5], Giuseppe Surico[6], Pier Luigi Nimis[7], Antonio Evidente[1]

1 Department of Chemical Sciences, University of Naples "Federico II", Complesso Universitario Monte Sant'Angelo, Via Cintia 4, 80126 Napoli, Italy; antonio.evidente@unina.it (A.E.)
2 Allelopathy Group, Department of Organic Chemistry, Institute of Biomolecules (INBIO), School of Science, University of Cadiz, C/Republic Saharaui, s/n, 11510, Puerto Real, Cadiz, Spain
3 Institute for Sustainable Plant Protection-CNR, Unit of Bari, via G. Amendola 122/d, Bari 70126, Italy. trifone.daddabbo@ipsp.cnr.it (TD)
4 Department of Molecular Medicine and Medical Biotechnologies, University of Naples Federico II, Via Pansini 5, 80131 Napoli, Italy; emanuela.roscetto@unina.it (E.R.); mariarosaria.catania@unina.it (M.R.C.); chiaravarriale98@gmail.com (C.V.)
5 Institute of Sciences of Food Production Sciences, National Research Council, Via Amendola 122/O, 70125 Bari, Italy; mariachiara.zonno@ispa.cnr.it (M.C.Z.); claudio.altomare@ispa.cnr.it(C.A.)
6 Department of Agriculture, Food, Environment, and Forestry (DAGRI), Section of Agricultural Microbiology, Plant Pathology and Enthomology, University of Florence, Italy Italy giuseppe.surico@unifi.it (G.S:)
7 Department of Life Sciences, University of Trieste, Via L. Giorgieri 10, 34127 Trieste, Italy; nimis@units.it (P.L.N.)
* Correspondence: e-mail: jesus.zorrilla@uca.es (J.G.Z.)

Index

Figure S1. ^{1}H NMR spectrum (400 MHz) of (+)-usnic acid (**1**) in $CDCl_3$ 3

Figure S2. ESI MS spectrum of (+)-usnic acid (**1**) recorded in positive mode 3

Figure S3. ^{1}H NMR spectrum (500 MHz) of methyl orsellinate (**2**) in $CDCl_3$ 4

Figure S4. ESI MS spectrum of methyl orsellinate (**2**) recorded in positive mode 4

Figure S5. ^{1}H NMR spectrum (500 MHz) of orcinol (**3**) in $CDCl_3$ 5

Figure S6. ESI MS spectrum of orcinol (**3**) recorded in positive mode 5

Figure S7. ^{1}H NMR spectrum (400 MHz) of (+)-montagnetol (**4**) in CD_3OD 6

Figure S8. ESI MS spectrum of (+)-montagnetol (**4**) recorded in positive mode.. 6

Figure S9. ^{1}H NMR spectrum (400 MHz) of 4-cloroorcinol (**5**) in $CDCl_3$ 7

Figure S10. ESI MS spectrum of 4-cloroorcinol (**5**) recorded in positive mode 7

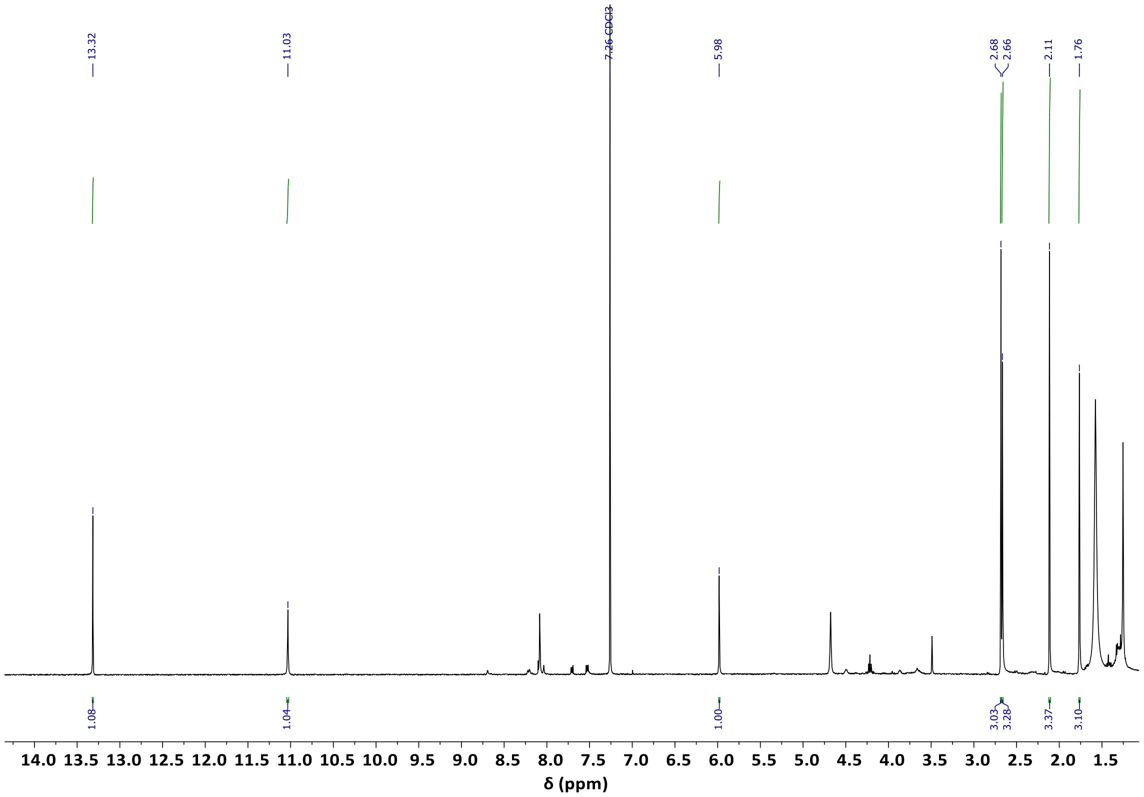

Figure S1. ^{1}H NMR spectrum (400 MHz) of (+)-usnic acid (**1**) in $CDCl_3$

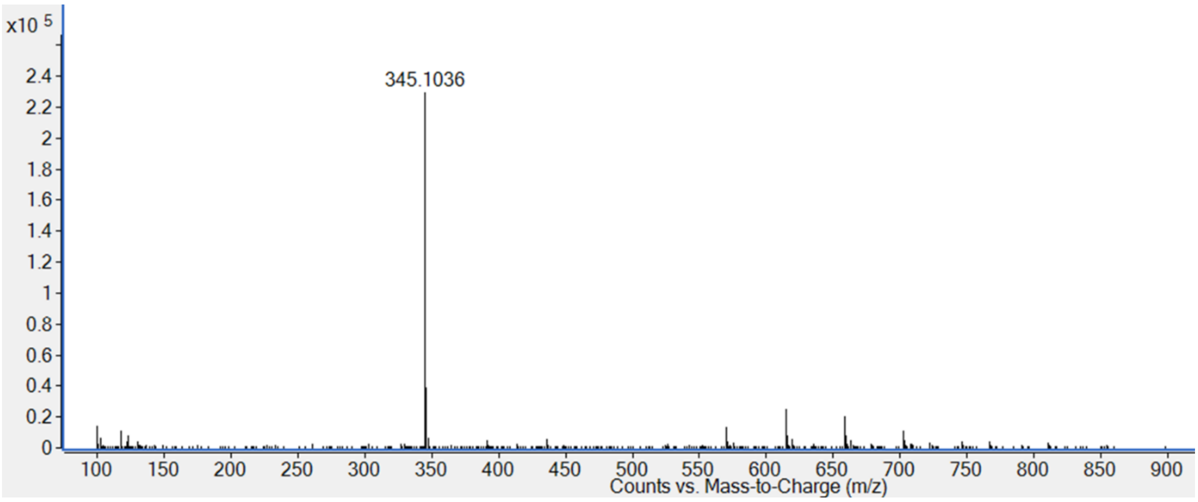

Figure S2. ESI MS spectrum of (+)-usnic acid (**1**) recorded in positive mode

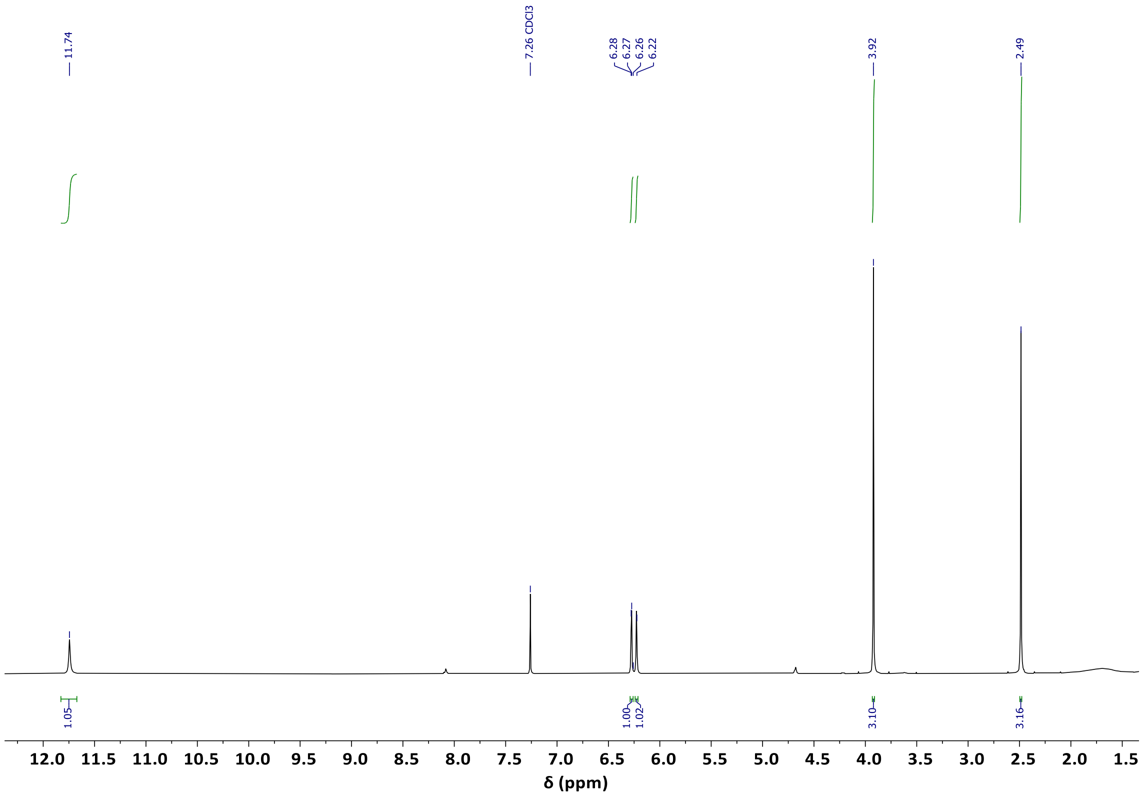

Figure S3. ^{1}H NMR spectrum (500 MHz) of methyl orsellinate (**2**) in $CDCl_3$

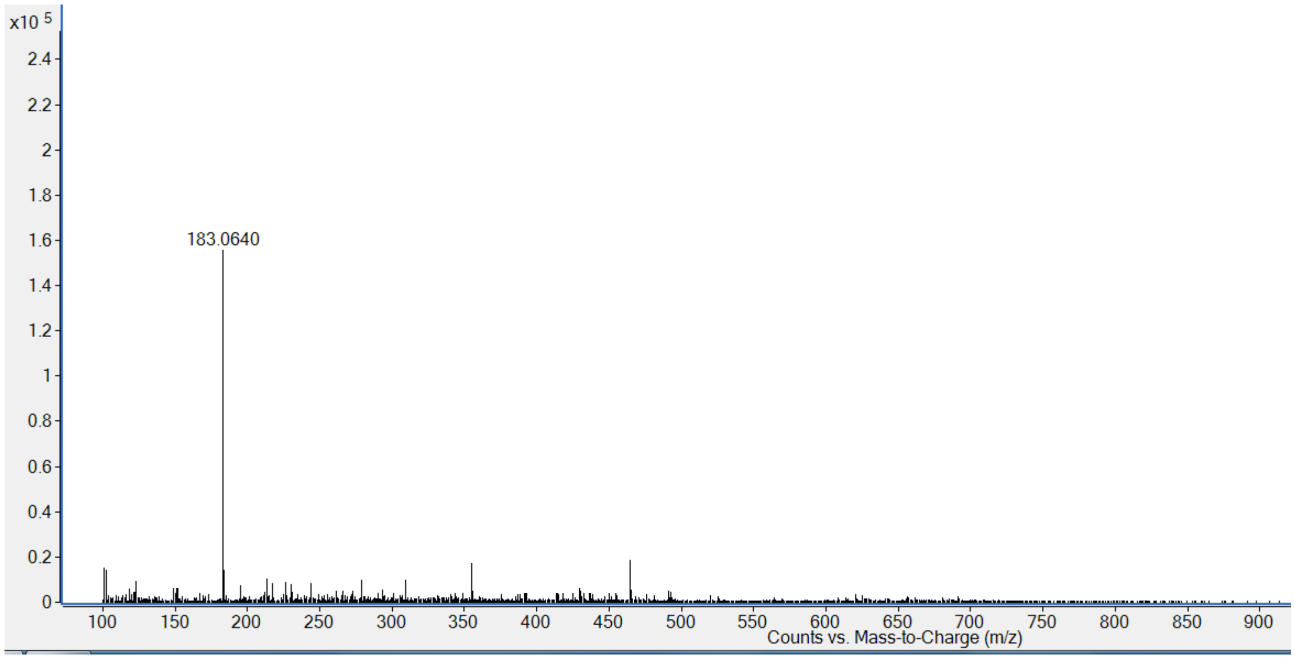

Figure S4. ESI MS spectrum of methyl orsellinate (**2**) recorded in positive mode

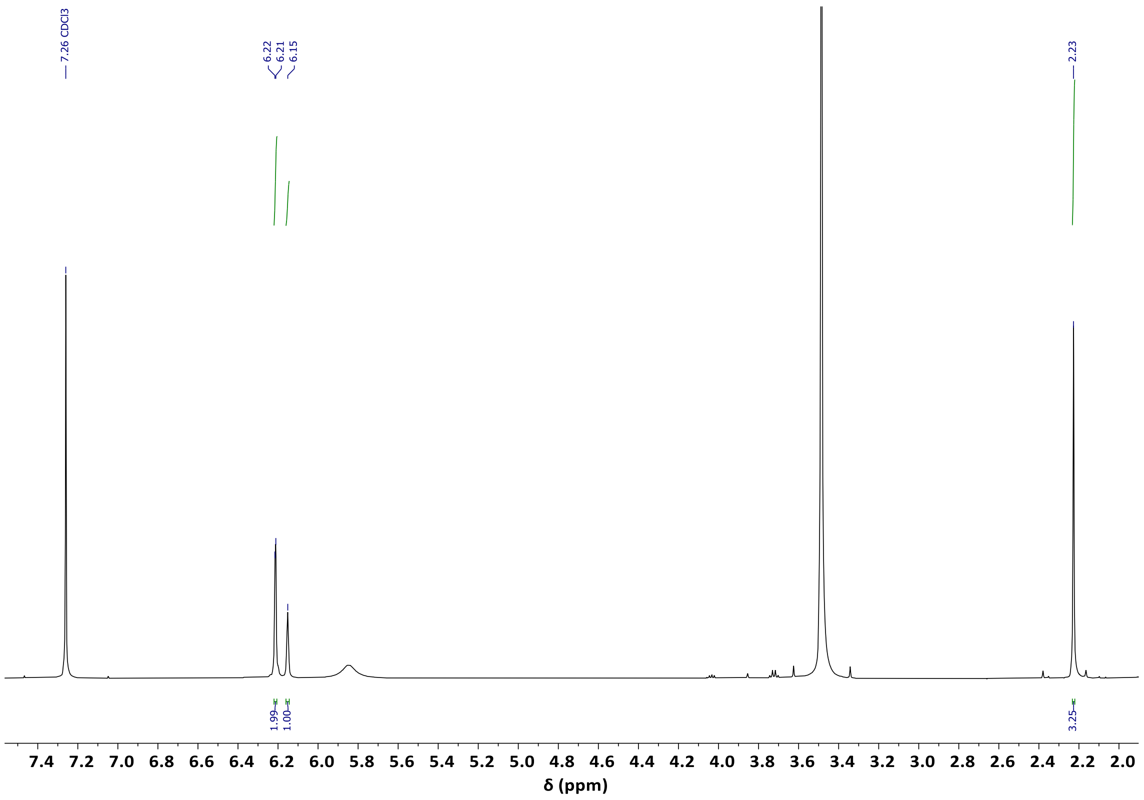

Figure S5. ^{1}H NMR spectrum (500 MHz) of orcinol (**3**) in $CDCl_3$

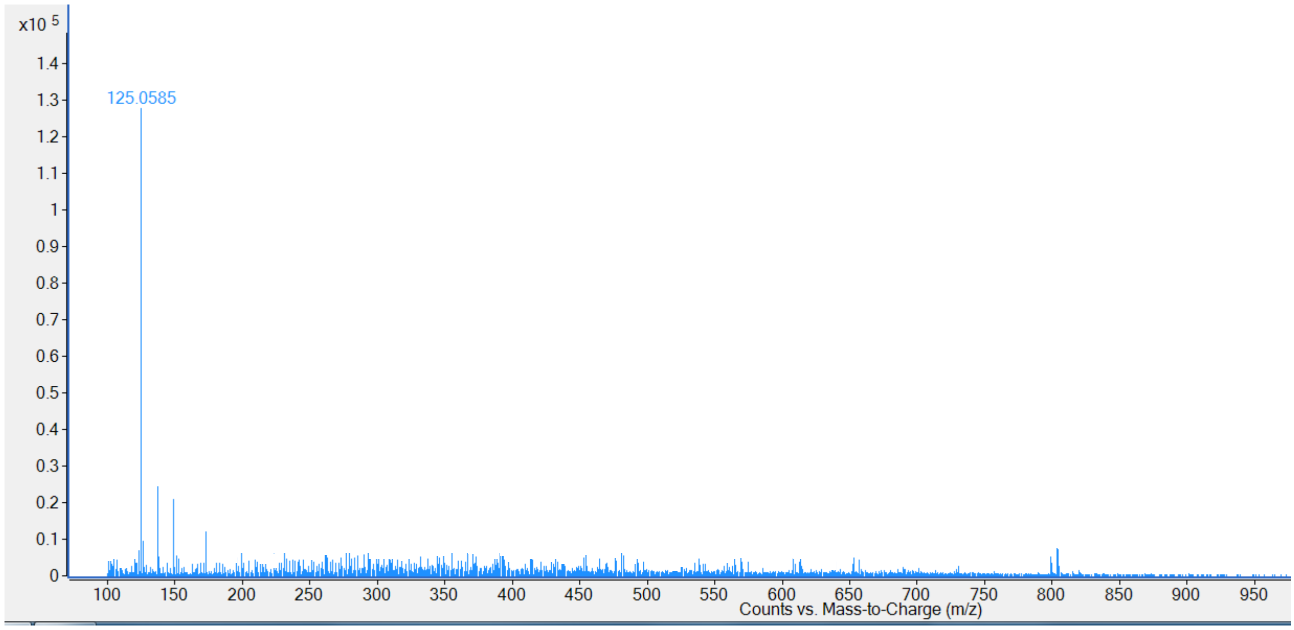

Figure S6. ESI MS spectrum of orcinol (**3**) recorded in positive mode

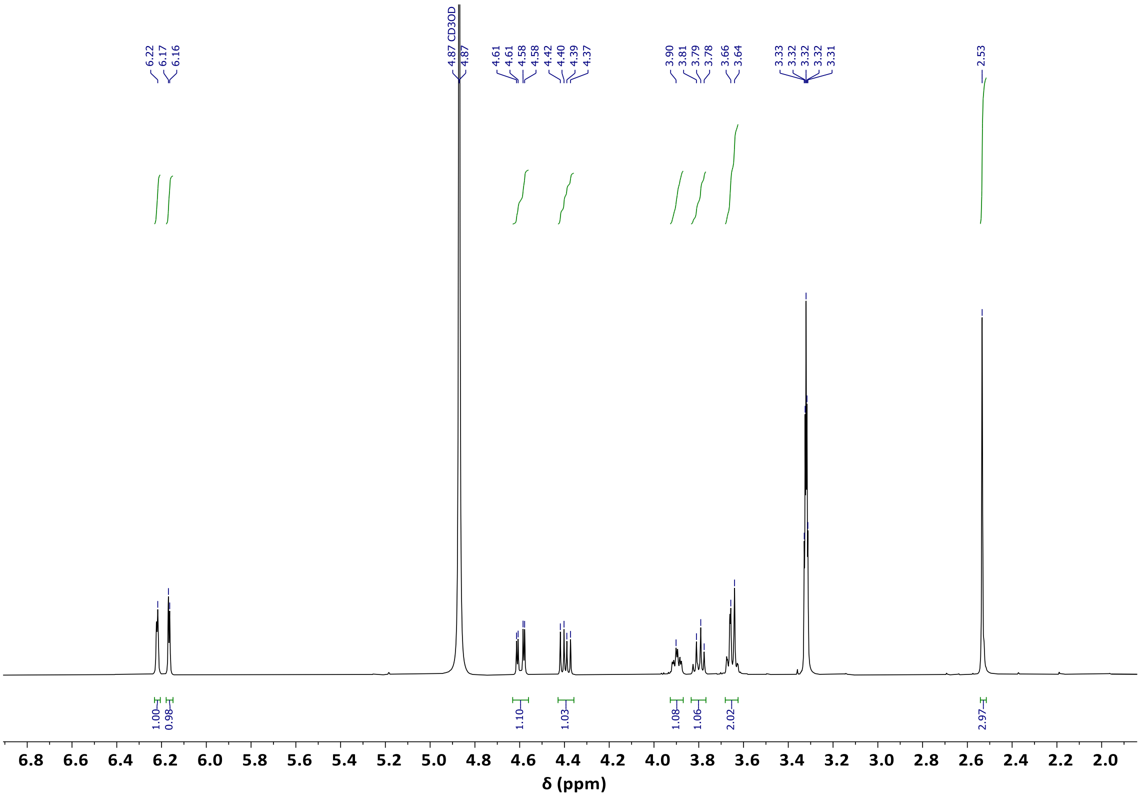

Figure S7. ^{1}H NMR spectrum (400 MHz) of (+)-montagnetol (**4**) in CD_3OD

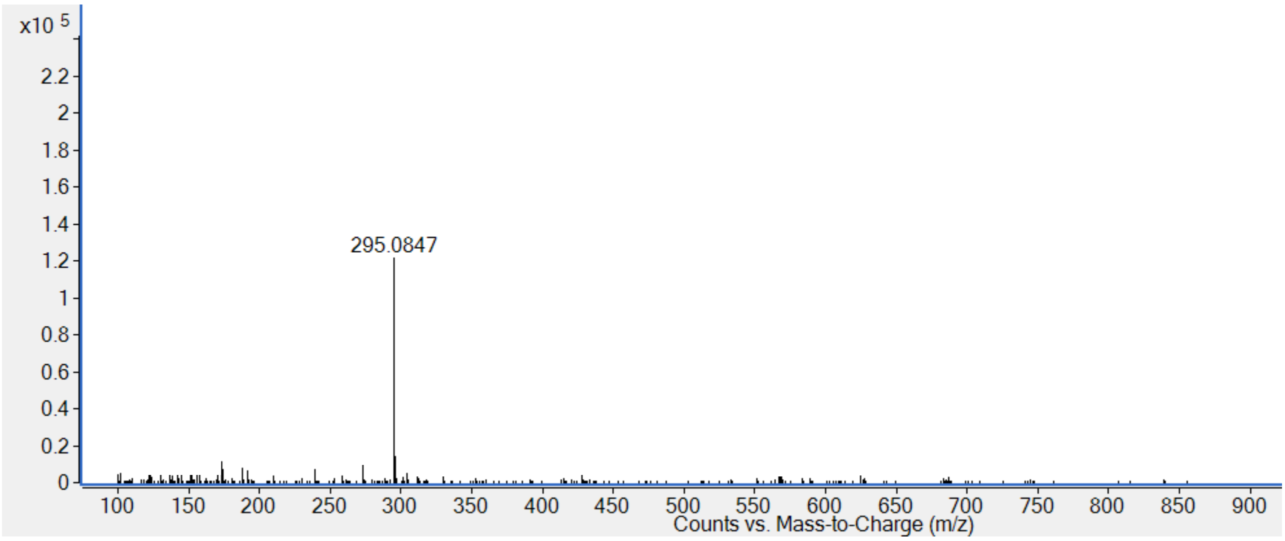

Figure S8. ESI MS spectrum of (+)-montagnetol (**4**) recorded in positive mode

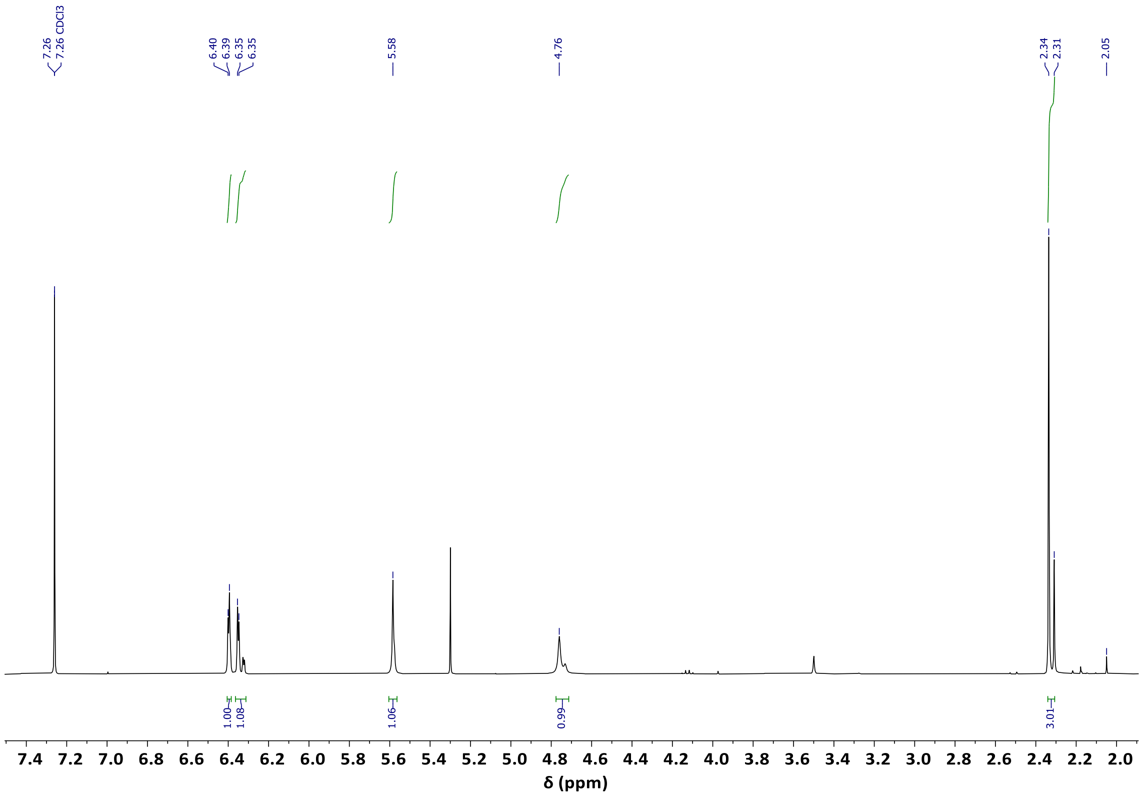

Figure S9. ^{1}H NMR spectrum (400 MHz) of 4-cloroorcinol (**5**) in $CDCl_3$

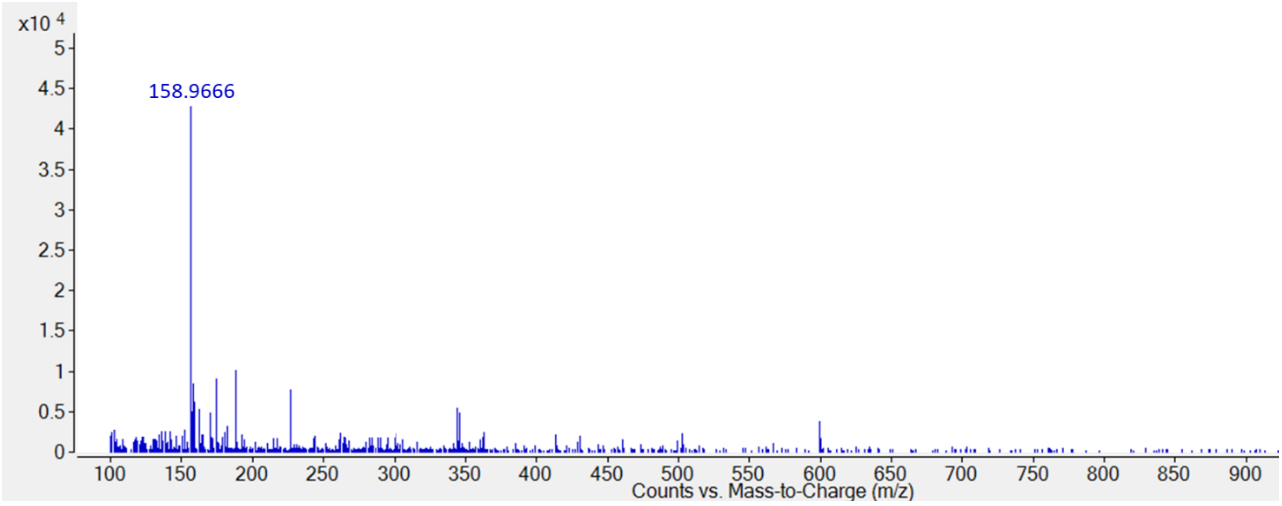

Figure S10. ESI MS spectrum of 4-cloroorcinol (**5**) recorded in positive mode
